# Neck swelling in a patient with gout - What is the cause?

**DOI:** 10.51866/tyk.537

**Published:** 2026-02-25

**Authors:** Siew Houy Chua

**Affiliations:** 1 Department of Internal Medicine, IMU University, Jalan Rasah, Bukit Rasah, Seremban, Negeri Sembilan, Malaysia.

**Keywords:** Gout, Tophi, Atypical presentations

## Abstract

We describe the case of a 55-year-old man with underlying tophaceous gout who presented with a 6-month history of multiple joint pains and associated constitutional symptoms. Physical examination revealed firm and non-tender swelling measuring 3x4 cm adjacent to the left clavicular head. This swelling did not move with deglutition. Neck imaging showed bilateral heterogeneous soft tissue lesions at the sternoclavicular, first costochondral and acromioclavicular joints. Multiple lytic lesions with cortical destruction involving the upper manubrium and distal ends of both clavicles were seen. Fine needle aspiration of the sternoclavicular joint yielded a small amount of synovial fluid, and its histopathological analysis confirmed the presence of monosodium urate crystal deposition. This case demonstrates a rare manifestation of tophaceous gout at the sternoclavicular joint, underscoring its ability to mimic other pathologies and the consequent challenges in diagnosis.

## Case summary

**Figure 1:**
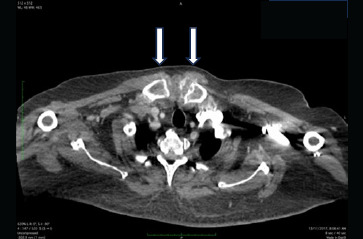
Axial view of the neck and thoracic CT scan showing bilateral soft tissue swelling at the head of the clavicles with calcification.

**Figure 2:**
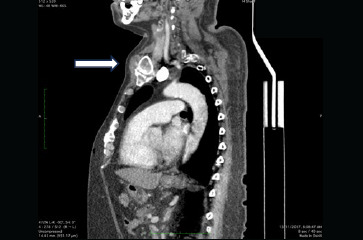
Sagittal view of the neck and thoracic CT scan showing soft tissue swelling at the sternoclavicular joint.

A 55-year-old man with underlying tophaceous gout presented with a 6-month history of chronic polyarticular joint pain, accompanied by unintended weight loss and difficulty swallowing. He denied fever, abdominal pain, change in bowel habits or melena. Upon physical examination, multiple tophi were observed at the metatarsophalangeal, elbow and hand joints, associated with generalised joint tenderness. Additionally, non-tender, firm swelling measuring 3x4 cm was noted adjacent to the left clavicular head, remaining immobile during swallowing. There was no abdominal mass or clinical evidence of hyperthyroidism. Other physical examination findings were also unremarkable. Laboratory investigations indicated raised inflammatory markers and serum uric acid levels, with normal thyroid function. Oesophagogastroduodenoscopy revealed a grade 3 oesophagitis according to the Los Angeles classification, but no upper gastrointestinal mass was found. Computed Tomography (CT) scans of the neck and thorax confirmed symmetrical, heterogeneous soft tissue lesions at both sternoclavicular joints (Figures 1 and 2), as well as the first costochondral and acromioclavicular joints. Multiple lytic lesions with cortical destruction were observed, involving the upper manubrium and distal ends of both clavicles. The thyroid gland exhibited no enlargement or focal lesions.

## Questions

1. What differential diagnoses would you consider at this point?
2. What further investigations should be carried out?

## Answers

1. What differential diagnoses would you consider at this point?
  - Malignancy: The presence of unintentional weight loss and bony lytic lesions raises concern for malignancies such as gastrointestinal cancer, lung cancer, multiple myeloma and sarcoma.
  - Osteomyelitis: Notably, osteomyelitis may produce bony lytic lesions that can mimic neoplastic growth.^1^
  - Inflammatory arthritis: The symmetrical distribution of the articular soft tissue lesions across the bilateral joints suggests the possibility of inflammatory arthritis, including conditions such as rheumatoid arthritis or gout.
2. What further investigations should be carried out?
  - Malignancy workup: Conduct a CT scan of the abdomen and pelvis to identify potential primary or metastatic lesions. Perform a laboratory workup to assess for indicators of multiple myeloma. Consider the possibility of a fluorodeoxyglucose-positron emission tomography scan at a later stage.
  - Bone biopsy: Undertake histopathological examination of the bone to detect evidence of both malignancy and infection.
  - Serology tests: Test for rheumatoid factor and anti-citrullinated protein antibody.
  - Bedside joint ultrasound: Perform an ultrasound of the sternoclavicular or acromioclavicular joints to look for the double contour sign, tophi, punctiform deposits in the synovial membrane and hyperechoic spots in the synovial fluid. A diagnosis of gout can be confirmed when monosodium urate (MSU) crystals are identified upon aspiration of the synovial fluid.^2^

## Case progress

Bedside ultrasound-guided fine needle aspiration of the sternoclavicular joint was conducted. It yielded a small amount of synovial fluid, where its histopathological examination revealed hypocellular smears containing numerous clusters and dispersed needle-shaped birefringent crystals, consistent with gouty crystals.

A barium swallow showed normal peristalsis of the oesophagus with a lateral oesophageal indentation attributed to cervical osteophyte, potentially contributing to his dysphagia. Further investigations ruled out malignancies.

The patient was diagnosed with an acute recurrent gout flare, with underlying chronic tophaceous gouty arthritis. This was characterised by structural joint damage and a significant decline in his overall health and quality of life. The neck swelling represented an atypical localisation of tophaceous gout.

He was treated with colchicine and corticosteroids for acute symptom relief, and his long-term gout management was optimised. At the 3-month follow-up, his polyarticular joint pain had markedly improved, with no evidence of new tophus formation. The long-term treatment goal is to maintain serum uric acid levels around 300 μmol/L to suppress gout flares and promote reduction in tophus size and number.

## Discussion

Chronic tophaceous gout classically occurs after 10 years or more of recurrent polyarticular gout. To date, there is no definitive explanation for the predilection of tophus formation at particular anatomical locations. Tophi are classically found in joints, tendons, ligaments and subcutaneous tissues. Nevertheless, atypical deposition has been reported in less common sites such as the head, neck, axial skeleton, viscera and even middle ear.^3,4^

When gout deposits occur at unusual sites, they often pose a diagnostic challenge, leading to delays in establishing the diagnosis and initiating appropriate treatment. The presence of associated constitutional symptoms may further complicate the clinical picture, necessitating the exclusion of serious conditions such as infection and malignancy before a definitive diagnosis can be made. Gout can also mimic tumourous conditions, with reports describing cases involving the skin or muscles resembling soft tissue tumours and spinal gout presenting similarly to metastatic disease.^5,6^ Sant and Dias reported a case of primary gout involving the sternoclavicular joint in an 18-year-old girl, which was initially treated as pyogenic arthritis before the diagnosis of acute gouty arthritis was confirmed.^7^Radiological evaluation plays an essential role in confirming gout, particularly in atypical or diagnostically challenging presentations. Characteristic imaging features include bone erosions with overhanging edges, sclerotic margins, bone proliferation, joint space narrowing and soft tissue masses (tophi), which may appear calcified on plain radiographs or CT scans. On ultrasonography, a tophus typically appears as an inhomogeneous mass with mixed echogenicity (ranging from hypoechoic to hyperechoic) surrounded by a thin anechoic rim. The characteristic double contour sign – a bright hyperechoic line overlying the dark hyaline cartilage surface, representing MSU crystal deposition – may also be observed. However, ultrasonographic detection of tophi or MSU crystals remains highly operator-dependent. Our case illustrates an uncommon presentation of tophaceous gout involving the sternoclavicular joint, manifesting as neck swelling that clinically mimicked more serious pathologies such as neoplastic or infectious disease. This underscores the importance of maintaining a high index of suspicion for gout in patients presenting with atypical joint or soft tissue lesions, particularly in those with hyperuricaemia or a history of gout. A comprehensive approach integrating clinical assessment, appropriate imaging and, where feasible, crystal analysis remains crucial for accurate diagnosis and optimal management.
